# A tribute to mentor and role model: Dr. Nicholas Lechmere Tilney

**DOI:** 10.3389/frtra.2026.1862909

**Published:** 2026-05-13

**Authors:** Jerzy W. Kupiec-Weglinski

**Affiliations:** The Dumont-UCLA Transplantation Center, Department of Surgery, Division of Liver and Pancreas Transplantation, David Geffen School of Medicine at UCLA, Los Angeles, CA, United States

**Keywords:** Brigham & Women's Hospital, cyclosporine, Harvard University, N.L. Tilney, Peter Bent Brigham Hospital, Boston, transplantation

**Figure 1:**
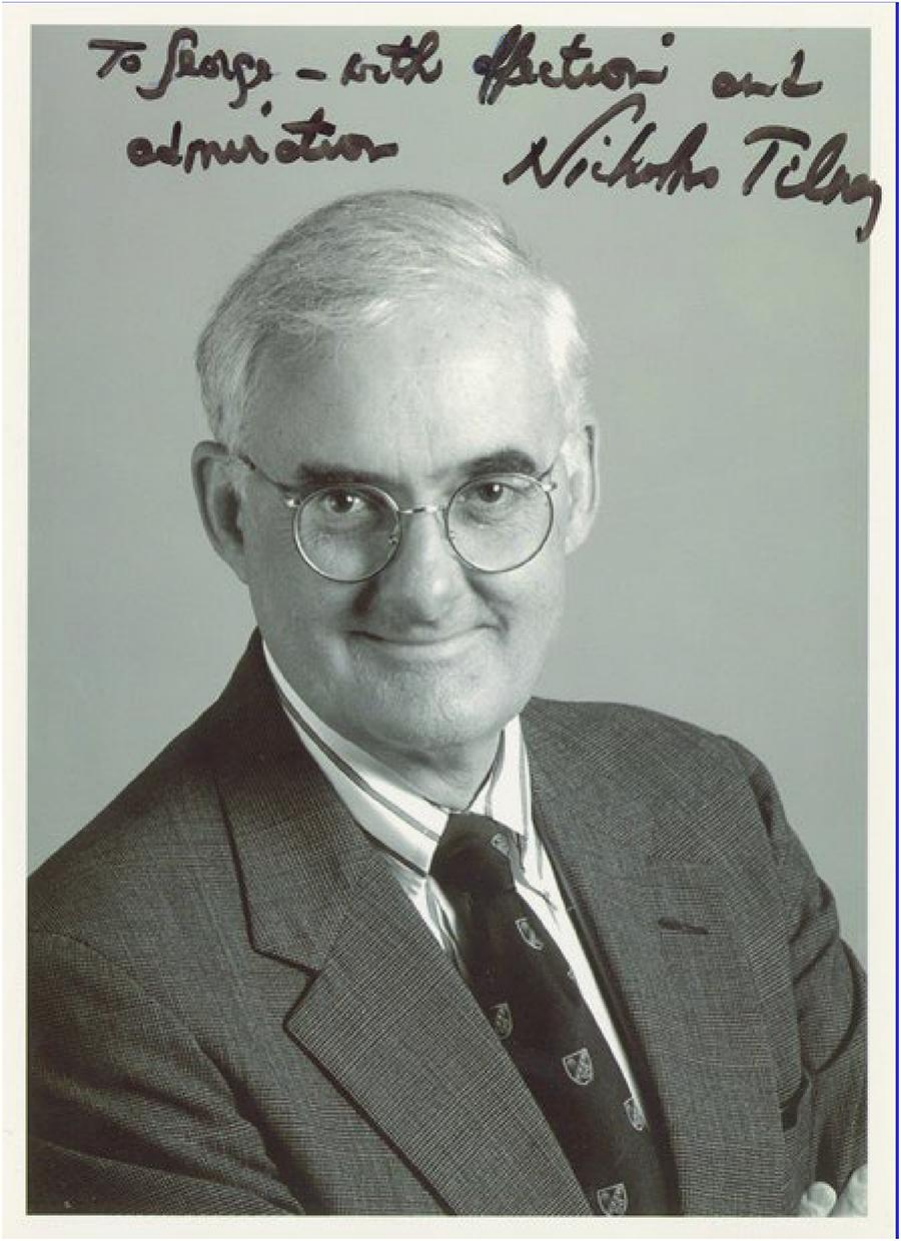
Nicholas L. Tilney (1935–2013).

Some mentors teach skills, while others shape the very way one thinks about science, medicine, and life. Nicholas Lechmere Tilney, M.D., belonged firmly to the latter category (Figure 1). To those of us fortunate enough to train under him at Harvard and to know him personally, he was not only a pioneering transplant surgeon-scientist but also a model of intellectual rigor, curiosity, warmth, and humanity. His life's work helped transform organ transplantation from an uncertain experimental endeavor into a disciplined, life-saving field of modern medicine. Yet beyond his remarkable scientific achievements, his character, generosity, and mentorship remain most vivid.

Nick Tilney's path to medicine was anything but narrow. Born in New York City to a family with deep New England roots, he attended Groton School before entering Harvard College, where he graduated in 1958 with an A.B. in Art History. He was not only a scholar but also an accomplished athlete, proudly captaining Harvard's winning heavyweight crew. This early blend of intellectual breadth and disciplined teamwork would shape his later career.

After earning his medical degree from Cornell University Medical College in 1962, he then began surgical training in Chicago and returned to Boston to train at the legendary Peter Bent Brigham Hospital under its renowned Surgeon-in-Chief, Francis D. Moore. There, Nick encountered the groundbreaking work of the future Nobel Prize laureate Joseph E. Murray, whose landmark 1954 kidney transplant between identical twins marked the dawn of clinical organ transplantation. That moment and the intellectual environment surrounding it profoundly shaped Dr. Tilney's career.

From the beginning, he was captivated not only by the surgical challenges of transplantation but also by the deeper biological questions - how the immune system recognizes and rejects foreign tissue and how that response might be controlled. This dual commitment to clinical excellence and scientific inquiry defined him as one of the rare true surgeon-scientists of his generation (1).

After serving as a surgeon in the U.S. Navy during the Vietnam War, Dr. Tilney expanded his scientific training abroad. He first worked at Oxford's Sir William Dunn School of Pathology under James L. Gowans, a pioneer in cellular immunology, and later continued in Glasgow, deepening his understanding of transplantation biology. These formative years abroad not only sharpened his scientific thinking but also contributed to the distinctive style many later associated with him - an intellectual elegance, occasionally accompanied by a playful imitation of British formality.

Returning to Boston, Dr. Tilney completed his surgical training and joined the Harvard faculty in 1973. Within a few years, he became Director of the Transplant Service at what would become Brigham and Women's Hospital and Director of the Surgical Research Laboratory at Harvard Medical School. In 1992, he was named the inaugural Francis D. Moore Professor of Surgery, a fitting recognition of his contributions.

Scientifically, Dr. Tilney's impact was profound. At a time when transplant outcomes were constrained by high mortality and graft failure, he played a critical role in advancing immunosuppressive therapy. He was among the first to investigate and apply cyclosporine in clinical transplantation - a breakthrough that dramatically reduced rejection rates and transformed patient survival (2). What once required massive, nonspecific immunosuppression with devastating side effects became a more precise and manageable process.

But Dr. Tilney did not stop at clinical applications. Much of his early research focused on the cellular mechanisms underlying acute transplant rejection. By developing techniques to isolate graft-infiltrating cells, he and his colleagues characterized lymphocyte populations, their interactions, and the cytokine networks that drive rejection (3–6). These studies helped define the field of modern transplantation immunobiology.

As acute rejection became better controlled, Nick turned his attention to the persistent problem of chronic rejection. He was among the first to highlight its importance and to explore its underlying mechanisms, including antigen-independent factors such as ischemic injury (7) and the physiological consequences of brain death in organ donors. His work helped shift the field's focus toward long-term graft survival, a focus that remains central today (8–10).

Throughout his career, Dr. Tilney's laboratory was exceptionally productive. Supported by NIH funding for nearly three decades, he trained more than 40 research fellows from around the world, many of whom became leaders in transplantation. He authored more than 550 scientific papers and chapters, leaving a body of work that continues to shape the field.

In his later years, Nick increasingly turned to writing, producing a series of thoughtful, widely read books, including *Transplant: From Myth to Reality*, *A Perfectly Striking Departure*, and *Invasion of the Body*. These works reflected not only his scientific knowledge but also his deep appreciation for the history and philosophy of medicine.

Dr. Tilney was also deeply engaged with the broader transplant community, serving as president of the American Society of Transplant Surgeons and The Transplantation Society, and as chairman of the New England Organ Bank. Alongside these leadership roles, he was a strong early advocate against organ trafficking and transplant commercialism, working to uphold ethical standards in transplantation worldwide. His contributions were recognized with numerous honors, including the Roche Pioneer Award and the Roche Distinguished Achievement Award.

Yet for all these accomplishments, what set Nick apart was not only what he did but also how he did it. He was, above all, a gentleman - warm, approachable, and deeply humane. He had an extraordinary ability to put complex problems into perspective and to remind those around him of the larger purpose of their work.

He was also known for his individuality and quiet humor. Stories circulated about him taking a call as a senior resident while rowing on the Charles River - an image that perfectly captures his blend of discipline, independence, and understated confidence. At conferences, while others commented on the elaborate tones of British speakers, Dr. Tilney would occasionally adopt a similar cadence, delivering his remarks with a subtle wit that always distinguished him.

I arrived in Boston around Halloween in 1979, stepping into what was then the epicenter of transplantation research. The Peter Bent Brigham Hospital was, in every sense, a mecca. I will never forget my first Thanksgiving with Nick and his wife, Mary, in their beautiful Beacon Hill penthouse apartment, nor the many gatherings at their eighteenth-century country house in New Castle, New Hampshire. We often sailed together along the Piscataqua River and enjoyed leisurely lunches along the nearby coast. Evening lobster dinners, invariably accompanied by chilled chardonnay, brought friends, fellows, and families together in an atmosphere of warmth and ease.

**Figure 2:**
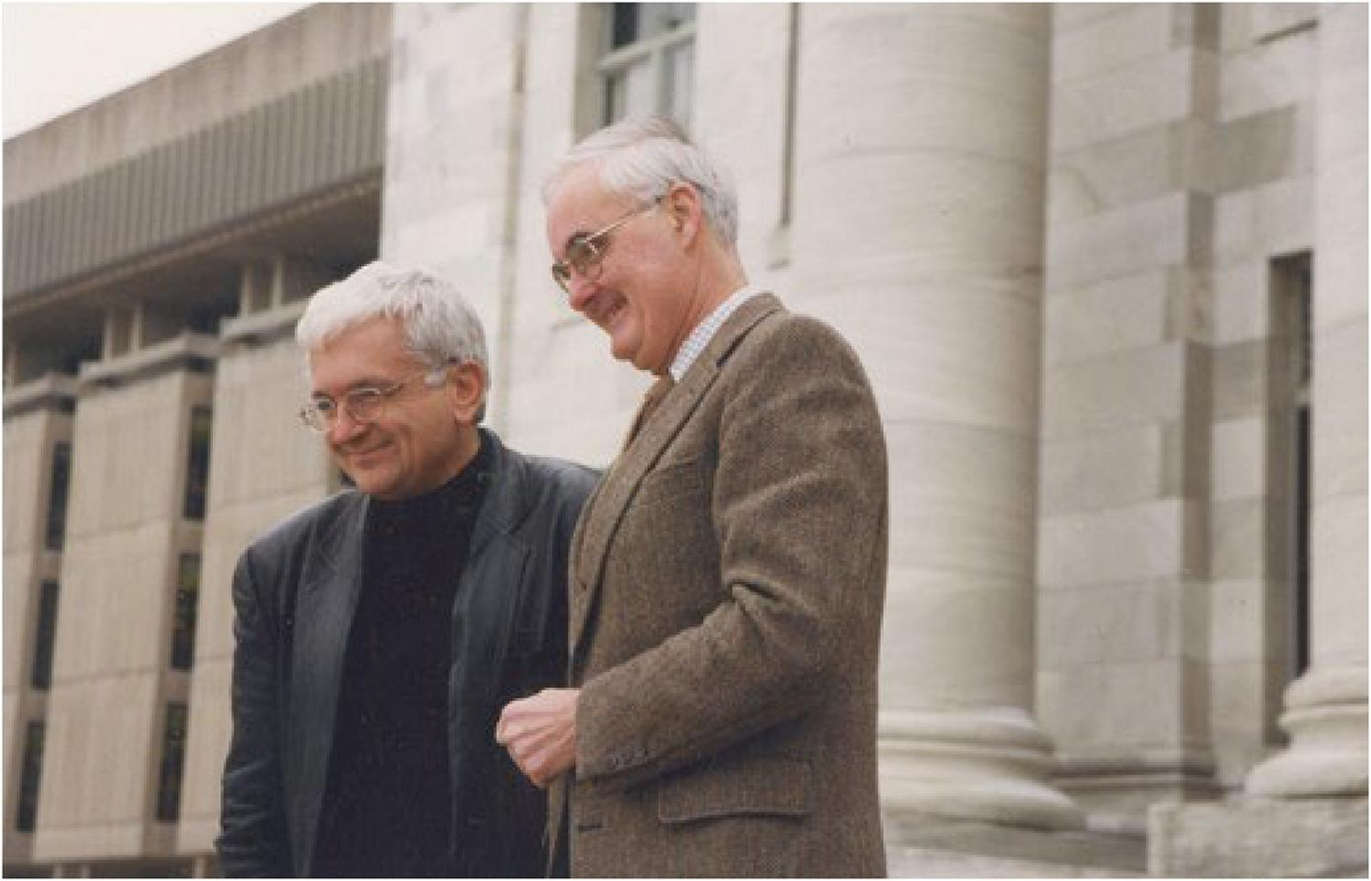
With Dr. Tilney, in front of Harvard Medical School, shortly before relocating to UCLA (May 1997).

These were not mere social occasions; they were extensions of a community that Nick and Mary, the Lab administrator, carefully cultivated. What began as a professional relationship soon grew into a lifelong friendship; over the years, our bond deepened far beyond the laboratory. In a gesture I have always cherished, Nick later became the godfather of my only daughter, Sophie, who is soon to become an anesthesiologist. In 1997, after 18 years at Harvard, I was recruited to UCLA to lead the scientific arm of one of the largest liver transplant programs in the U.S. under Dr. Ronald W. Busuttil, a step that reflected the foundation of training I had received from Dr. Tilney (Figure 2).

In the laboratory, Nick was an exceptional and generous mentor. He fostered a culture of logical thinking, precise writing, and genuine intellectual independence. Rather than imposing his ideas, he guided investigations with a light yet steady hand, enabling his trainees to develop their scientific voices and the confidence to pursue original thought. His mentorship extended beyond experiments and manuscripts; it shaped how we approached problems, questioned assumptions, and communicated science with clarity and integrity.

**Figure 3:**
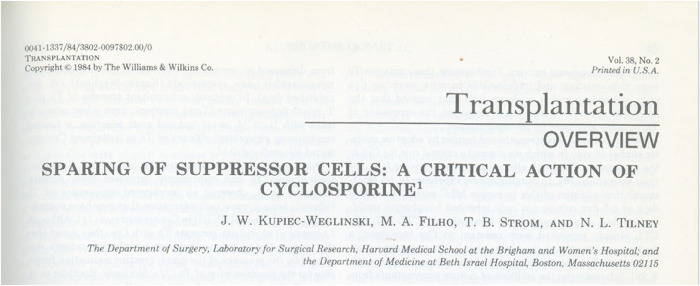
One of the first review articles on a new immunosuppressant – cyclosporine (Transplantation 1984; 38:97–101).

In the early 1980s, Nick's Lab at Harvard was among the few in the United States with access to a “magic powder” called cyclosporine, recently discovered by Jean-François Borel at Sandoz Laboratories in Switzerland. The excitement surrounding this new immunosuppressive agent was palpable, as it opened entirely new possibilities for transplantation. Working in that environment, at the forefront of both clinical and experimental innovation, profoundly shaped the trajectory of my career (Figure 3). Equally important were the close collaborations that Nick actively encouraged. Interactions with Harvard-based transplant pioneers, such as Terry Strom and Bernie Carpenter, fostered by Tilney's deeply held belief in collaboration over competition, led to a substantial and meaningful body of work. Those years were marked not only by productivity but also by a shared sense of purpose, and the publications that emerged from that period continue to stand as a testament to a uniquely creative and intellectually vibrant time.

In his final years, Nick faced a long illness with the same quiet courage and dignity that defined his life. He died on March 13, 2013, at his home in Boston, with Mary by his side. The surviving daughters - Rebecca, Louise, Victoria, and Frances - and his grandchildren, the youngest of whom, Nicholas, was born just days before Nick's passing, carry his legacy forward in deeply personal and profound ways.

The most enduring memories are often the most personal - and it is these memories that compel me to write this tribute many years after Dr. Tilney's passing. I do so at a reflective moment in my life, as I approach my 75th birthday and the later stages of my career. Such milestones inevitably invite a pause - an opportunity to look back not only at the trajectory of one's work but also at the people who gave it direction and meaning. Over the years, I have come to appreciate that the true measure of a career lies in the combination of personal accomplishments and the influence of those who shape how we think and act. To speak of Nick - my mentor and role model - is to speak not only of scientific discovery, but also of a philosophy of medicine. Looking back across decades in transplantation, I recognize ever more clearly how his approach to science, intellectual discipline, and humanity became a quiet standard by which I have measured my own path. For me, his legacy is not abstract - it is personal, enduring, and present in the way I continue to approach science and mentorship. If anything I have achieved carries meaning, it is in no small part because of the foundation he provided.
